# Rhegmatogenous retinal detachment induces more severe macular capillary changes than central serous chorioretinopathy

**DOI:** 10.1038/s41598-022-11062-6

**Published:** 2022-04-29

**Authors:** Junyeop Lee, Eoi Jong Seo, Young Hee Yoon

**Affiliations:** 1 Department of Ophthalmology, Asan Medical Center, University of Ulsan College of Medicine, 88 Olympic-ro 43-gil, Songpa-gu, Seoul, 138-736 South Korea; 2 Department of Ophthalmology, College of Medicine, Chungbuk National University Hospital, Chungbuk National University, 776 Sunhwan-1-ro, Seowon-gu, Cheongju, 28644 South Korea

**Keywords:** Prognostic markers, Retinal diseases

## Abstract

To investigate hemodynamic changes in macula-off rhegmatogenous retinal detachment (RRD) and its impact on visual prognosis by comparing with central serous chorioretinopathy (CSC). Using optical coherence tomography angiography (OCTA), vascular density in the superficial capillary plexus and deep capillary plexus (DCP) was retrospectively compared with that in contralateral unaffected eyes in macula-off RRD and CSC eyes. In RRD eyes, pre- and postoperative ultra-widefield (UWF) fluorescein angiography (FA) were obtained to analyze vascular changes. In OCTA, both macula-off RRD and CSC eyes showed less density in macular DCP, compared to the unaffected fellow eyes. Compared to CSC, eyes affected by macula-off RRD showed a reduction in DCP vascular density and an increase in foveal avascular zone area, although it had a much shorter macular detachment period. In macula-off RRD, less density of DCP was strongly correlated with longer duration of detachment, greater ellipsoid zone disruption, and poor visual recovery. In UWF-FA, detached retina showed capillary hypoperfusion, venous stasis and leakage, which were improved after reattachment. In conclusion, macular capillary loss of flow, which was associated with photoreceptor disruption, correlated with duration of detachment in RRD. Early reattachment and reperfusion are required for minimizing macular vasculature and photoreceptor damage in macula-off RRD.

## Introduction

Rhegmatogenous retinal detachment (RRD) is a retinal disease that can deteriorate central vision when it involves the macula. Prompt reattachment is generally required to prevent the involved eye from further photoreceptor damage and visual loss. Central serous chorioretinopathy (CSC) is also associated with detachment of the macula, often with a prolonged duration. However, it is associated with a better visual prognosis than macula involving RRD^1^. It has been postulated that photoreceptor cell apoptosis is the main reason for vision loss in RRD^2,3^. Still, it is not clear what exactly causes a difference in visual prognosis between macula-off RRD and CSC.

A number of studies have sought to elucidate the pathological mechanism of photoreceptor damage in RRD^4–13^. Peripheral nonperfusion and subsequent neovascularization were reported in some chronic RRD cases, indicating that detachment of the retina can cause retinal perfusion impairment^4–8^. Recent studies using optical coherence tomography angiography (OCTA) revealed that macular capillary perfusion was damaged even after macular reattachment^9–13^. In CSC, however, macular vascular alteration was minimal even after a longer duration of macular detachment^14,15^.

Therefore, we hypothesized that altered dynamics of retinal blood flow in RRD can reduce retinal perfusion, cause macular photoreceptor ischemia, and eventually deteriorate central vision. Using ultra-widefield (UWF) fluorescein angiography (FA) and OCTA, we analyzed both peripheral and central retinal circulation alterations/impairments in detached and reattached retina. We investigated the correlation between retinal hemodynamic changes in macula-off RRD and postoperative visual recovery. For comparison, vascular alterations in CSC were also studied.

## Methods

### Patients

This retrospective study adhered to the tenets of the Declaration of Helsinki and was approved by the Institutional Review Board and Ethics Committee of Yeungnam University Hospital and Asan Medical Center. An exemption was granted from the requirement for informed consent because the present study was retrospective research. All the methods were performed in accordance with relevant guidelines and regulations. Fifty-two eyes of 52 patients who were diagnosed with primary RRD at Yeungnam University Hospital and Asan Medical Center from March 2017 to December 2018, and underwent successful operation, including vitrectomy or scleral buckling, were included and analyzed. Retinal detachment occurring due to retinal holes, tears or dialysis was considered primary RRD, and tractional/exudative retinal detachments were excluded. We only included relatively acute, uncomplicated primary RRD that had a disease onset of less than 1 month and showed successful reattachment after the primary operation. Macular involvement in retinal detachment was defined as macula-off RRD, and this terminology is used throughout the manuscript.

For the comparison analysis, 42 eyes with CSC were also collected. Eyes showing subretinal fluid without any definite retinal break and focal/diffuse dye leakage on FA with choroidal hyperpermeability on indocyanine green angiography were defined as having CSC.

Highly myopic eyes over -6 diopters or the presence of concomitant retinal vascular diseases such as diabetic retinopathy, retinal vein or artery occlusion, Coats’ disease and familiar exudative vitreoretinopathy were considered exclusion criteria. Eyes presenting with retinal or choroidal vascular disease in the fellow eye were also excluded because the fellow eye was analyzed as a control group. We also excluded secondary macular morphological changes, including Irvine-Gass cystoid macular edema, epiretinal membrane, and macular holes, which can occur postoperatively and can influence OCTA segmentation. All patients were asked about their underlying systemic illnesses, such as diabetes mellitus or hypertension, and underwent a comprehensive eye examination, including best-corrected visual acuity (BCVA), slit-lamp biomicroscopy, color fundus photography, spectral domain optical coherence tomography (OCT), and UWF FA.

Surgical procedures were performed by experienced retinal surgeons (YHY and JL). Surgical decision-making and procedures were performed at the surgeons’ discretion. Operations were conducted in the operating room under general or retrobulbar anesthesia with aseptic preparation. Vitrectomy was performed with 25-gauge instruments (Constellation Vision System; Alcon Laboratories Inc., Fort Worth, TX, USA). All vitrectomies were performed as follows: core vitrectomy, induction of posterior vitreous detachment, relief of vitreoretinal traction around the retinal break, subretinal fluid drainage, endolaser photocoagulation, and tamponade with perfluoropropane gas or silicone oil. Scleral buckling was performed with a silicone sponge combined with cryotherapy around the retinal break.

### Optical coherence tomography

Spectral domain OCT (Spectralis; Heidelberg Engineering, Heldelberg, Germany) was performed at every patient visit to evaluate the status of retinal attachment and ellipsoid zone integrity. In RRD eyes, ellipsoid zone (EZ) integrity was graded according to the relative length ratio of the disrupted EZ in the single horizontal scan through the fovea: Grade 0: Intact EZ; Grade 1: less than 25% disruption; Grade 3: 25–50% disruption; and Grade 4: more than 50% disruption. In CSC eyes, the highest subretinal fluid (SRF) height at the fovea was measured manually using embedded software, which was provided by the manufacturer. SRF height was defined as the vertical distance from the tip of the RPE layer to the outer border of the detached retina at the fovea.

### Optical coherence tomography angiography

Twenty-six eyes with macula-off RRD and 42 eyes with CSC underwent OCTA. Data from healthy fellow eyes were obtained as a control. To avoid segmentation error, OCTA images were obtained after successful reattachment of RRD or complete SRF dry-up of CSC. A 3 × 3 mm area centered in the fovea was scanned with either AngioVue (Optovue Inc., Fremont, CA, USA) or AngioPlex (Zeiss Meditec Inc., Dublin, CA, USA). Both superficial capillary plexus (SCP) and deep capillary plexus (DCP) slab images were obtained to analyze the retinal capillaries. Inadequate quality of images of which signal strength index was below 50 in AngioVue or 6 in AngioPlex were excluded. The SCP was segmented with an inner boundary of 3 µm below the internal limiting membrane and an outer boundary of 15 µm below the inner plexiform layer (IPL). The DCP was segmented with an inner boundary of 15 µm below the IPL and an outer boundary of 70 µm below the IPL. In the case of incorrect segmentation, we manually adjusted the boundary between the specific layers.

Then, vascular flow density and foveal avascular zone (FAZ) area were calculated from both capillary plexuses to quantify foveal vascular status using a previously described method with ImageJ software^16^. Relative density (ratio to normal fellow eye) was used to calculate vascular flow density results because two different OCTA devices were used to measure vascular flow density in the present study.

In the preliminary study, only age had a significant correlation with the DCP vascular density of CSC among the variables, including duration of macular detachment, refractive errors, SRF height and anti-vascular endothelial growth factor (VEGF) use, which were previously reported to be related to choroidal changes in CSC^17,18^. Therefore, comparing the results of CSC and RRD, age-matched groups were selected (42 eyes with CSC and 26 eyes with macula-off RRD) and analyzed to minimize age-related confounding factors.

### Ultra-widefield fluorescein angiography

UWF FA (Optos 200 MA/200Tx; Optos PLC, Dunfermline, Scotland, United Kingdom) was performed before the operation to evaluate retinal perfusion status in all 52 RRD eyes (100%). Angiographic abnormalities, including vascular tortuosity, venous dilation, diffuse paravascular leakage, peripheral nonperfusion, marginal leakage, capillary stasis, delayed arteriovenous transit time, focal nonperfusion and neovascularization elsewhere, were graded and analyzed by a retinal specialist (JL). After successful operation and reattachment of the retina, UWF FA was taken again in 46 eyes (88%). The angiographic abnormalities described above were graded again for comparison.

### Statistical analysis

All statistical analyses were performed using the Statistical Package for the Social Sciences (version 21.0; SPSS Inc., IBM Company, Chicago, IL, USA). Continuous variables are presented as the mean ± standard deviation. Comparisons between groups were evaluated using Student’s t-test, chi-square test, or Fisher’s exact test, as appropriate. In all analyses, a value of *p* < 0.05 was considered statistically significant.

### Conference presentation

Part of this study was presented at the Meeting of the Club Jules Gonin, Dubrovnik, Croatia, September 2020.

## Results

Of 52 RRD cases, 38 eyes had macula-off RRD at diagnosis. Among them, 26 eyes acquired postoperative OCTA and had a matched age distribution with CSC eyes. All 42 CSC eyes underwent OCTA at the resolution of SRF. SRF resolved spontaneously in nine eyes, while other eyes needed treatment (anti-VEGF for 27 eyes, anti-VEGF with focal laser for three eyes, and photodynamic therapy for three eyes) for SRF resorption. The duration of the macula-off period was significantly shorter in RRD cases (mean 6.3 days) than in CSC cases (mean 210.6 days) (Table 1). However, the final BCVA was significantly better in CSC eyes than in RRD eyes.

**Table 1:** Comparison of characteristics in macula-off rhegmatogenous retinal detachment and central serous chorioretinopathy.

	Macula-off RRD (n = 26)*	CSC (n = 42)	*P* value
Age (years)	51.5 ± 7.8	51.5 ± 8.9	0.985^a^
Male/female (eyes)	19/7	36/6	< 0.001^b^
Diabetes or hypertension (eyes)	4 (15%)	5 (12%)	0.680^b^
Duration of macular-off period (days)	6.3 ± 5.6	210.6 ± 129.2	< 0.001^a^
Visual acuity (logMAR)	0.43 ± 0.26	0.15 ± 0.19	0.040^a^
Refractive error (diopter)	− 0.59 ± 1.71	− 0.34 ± 1.66	0.562^a^
SRF height (μm)	Exceeded measurement limit	140.8 ± 127.8	

*CSC* central serous chorioretinopathy, *RRD* rhegmatogenous retinal detachment, *SRF* subretinal fluid.

*Macula-off RRD patients who underwent optical coherence tomography angiography.

^a^*P* values were calculated with independent *t*-test.

^b^*P* values were calculated with chi-square test.

**Figure 1:**
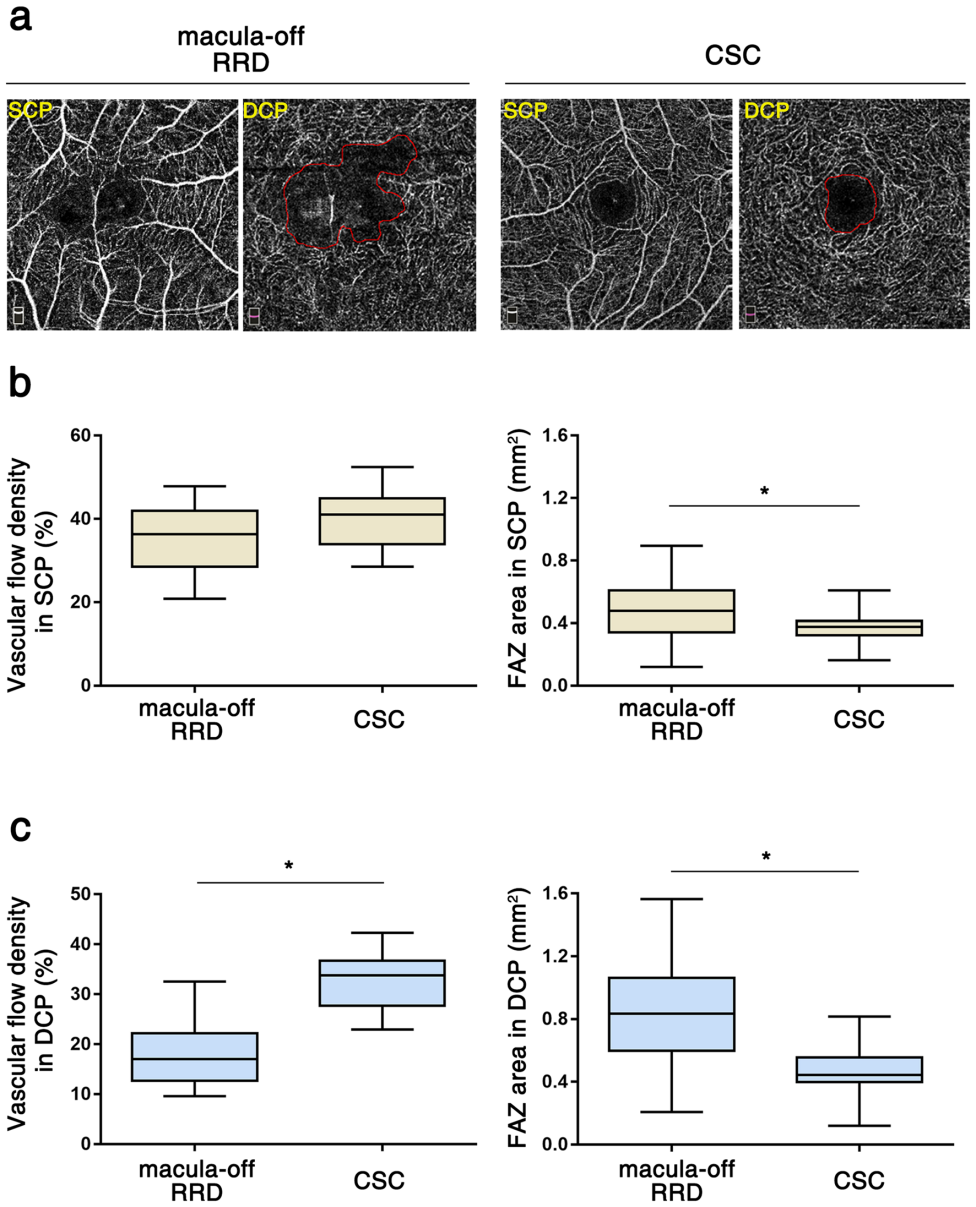
Macular perfusion state after reattachment in rhegmatogenous retinal detachment (RRD) and central serous chorioretinopathy (CSC). (**a**) Optical coherence tomography angiography results in both RRD and CSC eyes. Multiple dropouts in the macular capillary vessels in the deep capillary plexus (DCP) and an enlarged foveal avascular zone (FAZ) in both the superficial and deep capillary plexuses in RRD. In contrast, CSC shows well-preserved vascular flow density and small FAZ areas in both capillary plexuses. (**b**) In the superficial capillary plexus (SCP), vascular flow density was not different between RRD and CSC (*p* = 0.074). The FAZ area was significantly larger in RRD than in CSC (*p* < 0.003). (**c**) In the deep capillary plexus (DCP), vascular flow density was lower in RRD than in CSC (*p* < 0.001). The FAZ area was also larger in RRD than in CSC (*p* < 0.001).

On OCTA images taken after successful reattachment and complete SRF resolution, both macula-off RRD and CSC eyes showed decreased vascular flow density and enlargement of the FAZ area compared to their fellow eyes (Table 2). Mean interval between the reattachment of retina and OCTA examination was 57.5 ± 29.6 days in macula-off RRD. The DCP showed more prominent deterioration than the SCP in both conditions. In addition, vascular flow density was significantly lower and FAZ area was significantly larger in macula-off RRD eyes than in CSC eyes (Fig. 1, Table 2). This difference was also prominent in the DCP.

**Table 2:** Analysis of macular optical coherence tomography angiography results in macula-off rhegmatogenous retinal detachment (and fellow eye) and central serous chorioretinopathy (and fellow eye).

	Macula-off RRD	CSC	*P* value
**Superficial capillary plexus**			
Vascular flow density (%)	35.61 ± 7.85 (41.01 ± 7.16)*	39.93 ± 6.78 (41.93 ± 7.12)	0.074
FAZ area (mm^2^)	0.49 ± 0.19 (0.33 ± 0.10)*	0.37 ± 0.10 (0.34 ± 0.08)	0.003
**Deep capillary plexus**			
Vascular flow density (%)	18.26 ± 7.09 (36.92 ± 5.99)*	32.75 ± 5.42 (36.81 ± 5.60)*	< 0.001
FAZ area (mm^2^)	0.85 ± 0.34 (0.36 ± 0.10)*	0.48 ± 0.15 (0.39 ± 0.09)*	< 0.001

Values of the control group (normal fellow eyes) are presented in parentheses.

*RRD* rhegmatogenous retinal detachment, *CSC* central serous chorioretinopathy, *FAZ* foveal avascular zone.

*P values comparing RRD or CSC and the fellow eye < 0.05.

**Figure 2:**
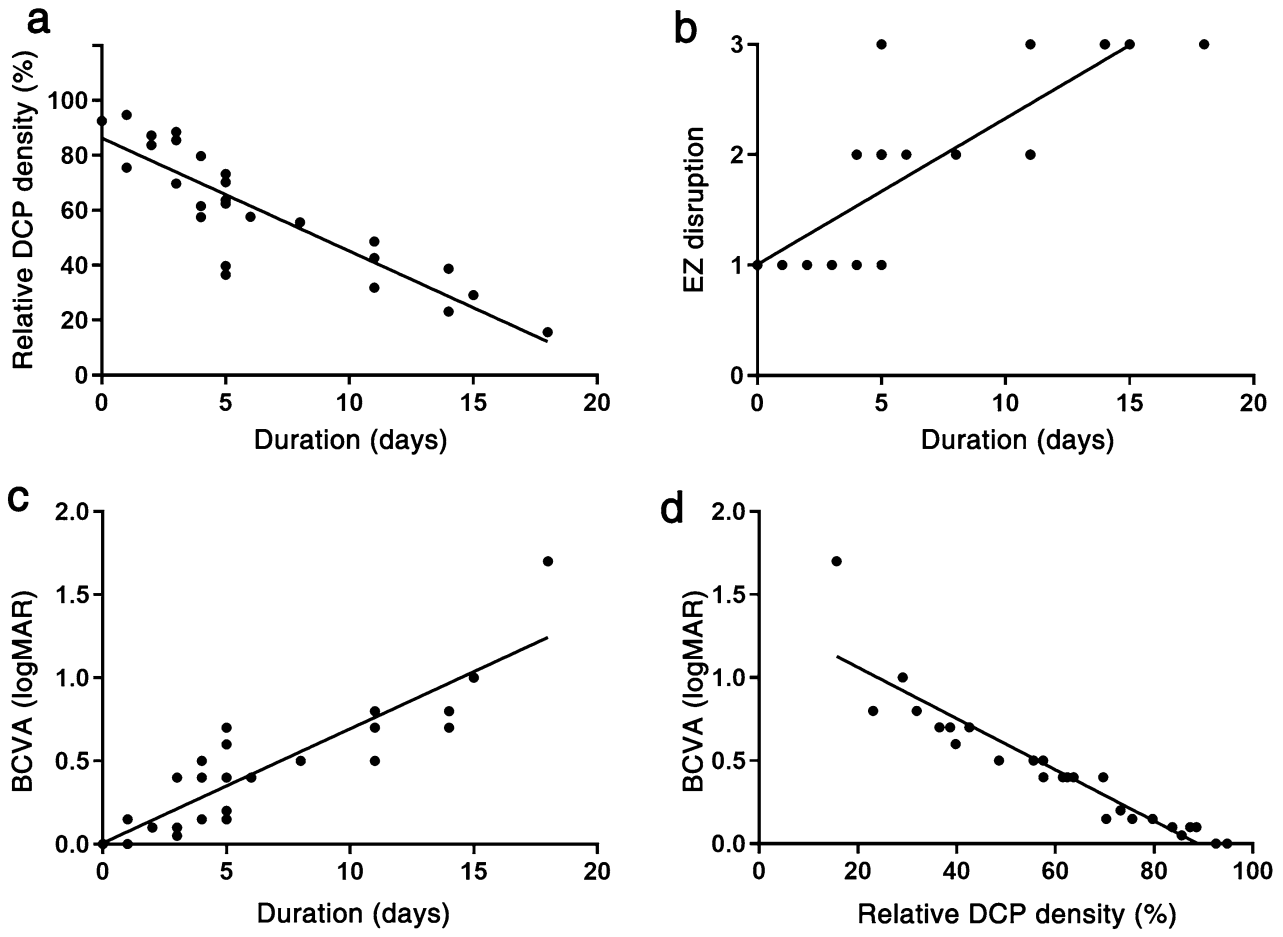
Association of optical coherence tomography angiography parameters and visual prognosis in relation to duration of detachment in macula-off rhegmatogenous retinal detachment. As the duration of macular detachment increased, the macular deep capillary plexus (DCP) density, ellipsoid zone (EZ) integrity, and visual acuity (VA) worsened. Eyes that could be reattached promptly showed good DCP density, EZ integrity and visual acuity (**a**–**c**). Relative DCP density (**a**) is defined that DCP proportion of the RRD eye compared to the normal control eye (fellow eye). DCP density was revealed to be strongly correlated with visual acuity (**d**).

**Figure 3:**
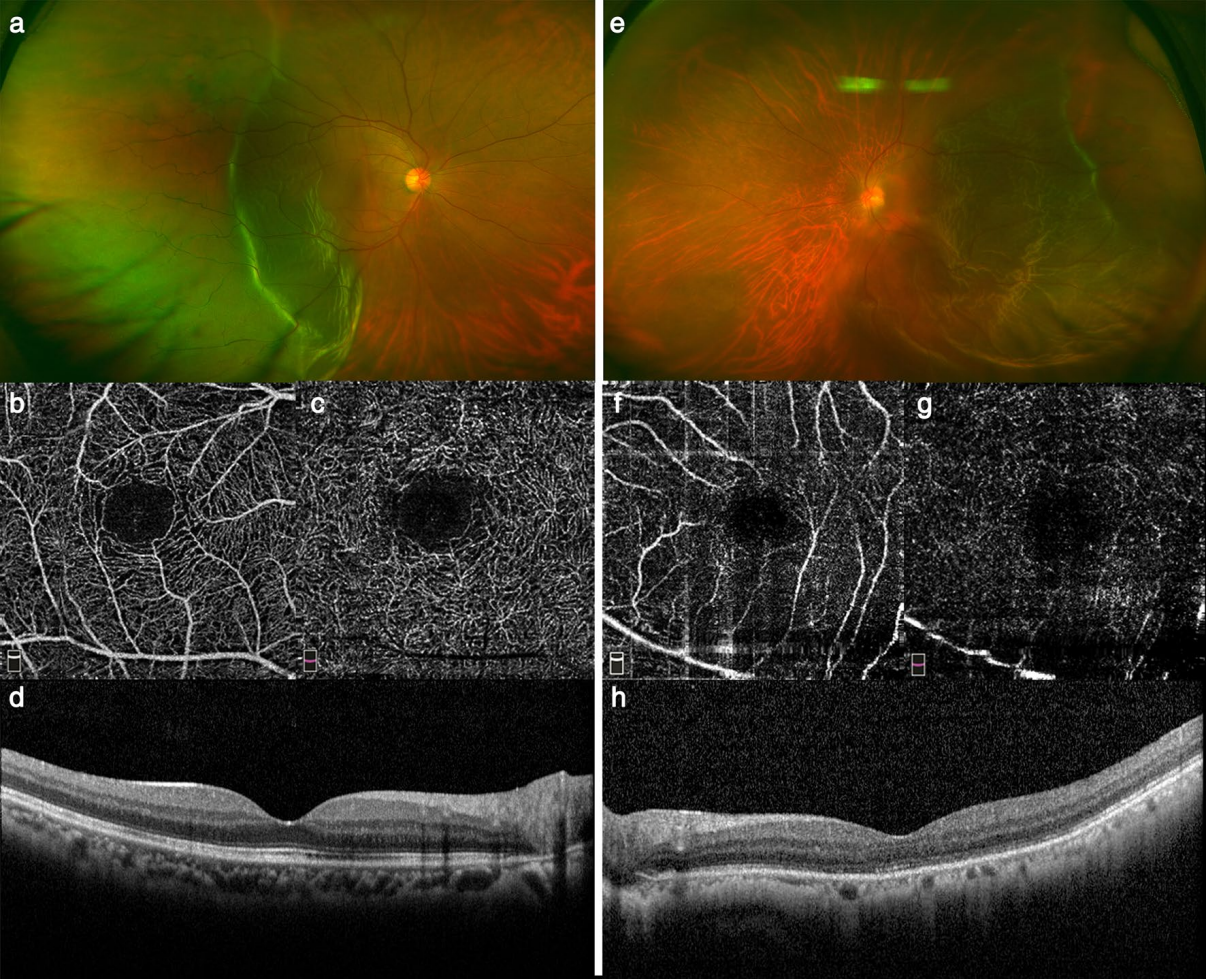
Representative case comparison between early and delayed reattachment after macula-off rhegmatogenous retinal detachment. Left column images (**a**–**d**) show images of retinal detachment with early reattachment (47 years old, initial vision of 20/1000, 2 days of detachment). Right column images (**e**–**h**) show images of retinal detachment with delayed reattachment (68 years old, initial vision of 20/630, 21 days of detachment). Both eyes underwent pars planar vitrectomy endolaser photocoagulation and perfluoropropane gas endotamponade. A wide color fundus photo (**a** and **e**) shows preoperative macular off rhegmatogenous retinal detachment. Postoperative optical coherence tomography angiography (**b**, **c** and **f**, **g**) images indicate better vascular density and a smaller foveal avascular zone area in early reattachment than in delayed reattachment. Early reattachment was associated with a good macular ellipsoid zone and a good final vision (20/25), while delayed reattachment was associated with a disrupted macular ellipsoid zone and a suboptimal final vision (20/100) (**d** and **h**).

Then, association between duration of detachment and the microvascular structure was examined. In the analysis of 26 eyes with macula-off RRD, DCP destruction was proportional to the duration of macular detachment (Fig. 2a). EZ integrity on OCT and final visual acuity were also proportional to the duration of macular detachment (Fig. 2b, c), supporting the positive correlation between DCP vascular density and final BCVA (Fig. 2d). In contrast, CSC eyes did not show a significant change in the macular capillary plexus or BCVA as the duration of macular detachment increased (See Supplementary Fig. S1). Representative case comparison was shown in Fig. 3.

**Figure 4:**
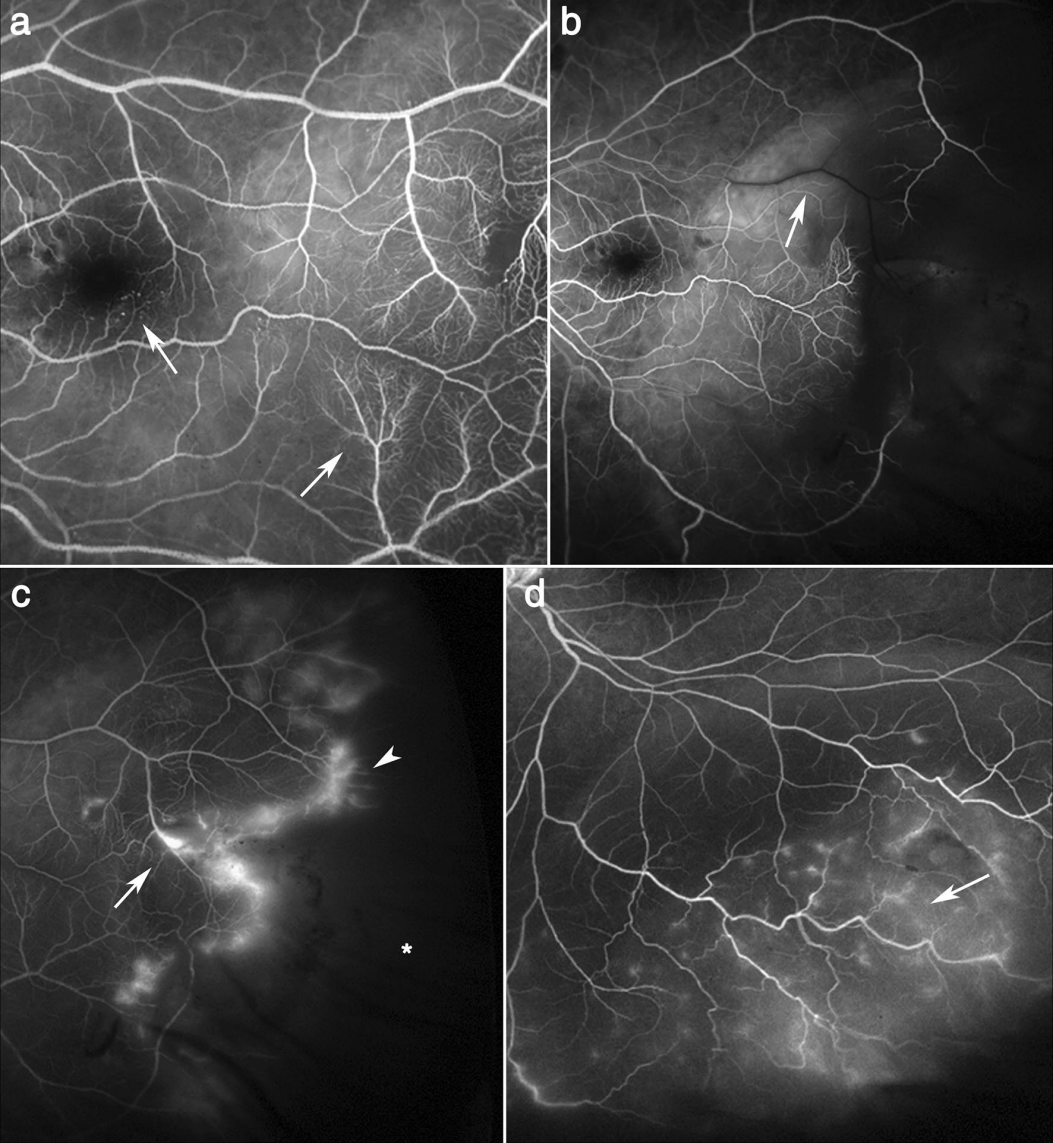
Representative images of perfusion impairment and vascular abnormalities detected in preoperative ultra-widefield fluorescein angiography in rhegmatogenous retinal detachment. Ultra-widefield fluorescein angiography was performed on eyes with rhegmatogenous retinal detachment before reattachment. Arrows indicate the vascular abnormalities that RRD eyes have. (**a**) Capillary stasis shown on the macula and periphery. (**b**) Delayed arteriovenous transit. (**c**) Neovascularization (arrow) marginal leakage (arrowhead) and peripheral nonperfusion (asterisk). (**d**) Vascular tortuosity and diffuse paravascular leakage.

**Figure 5:**
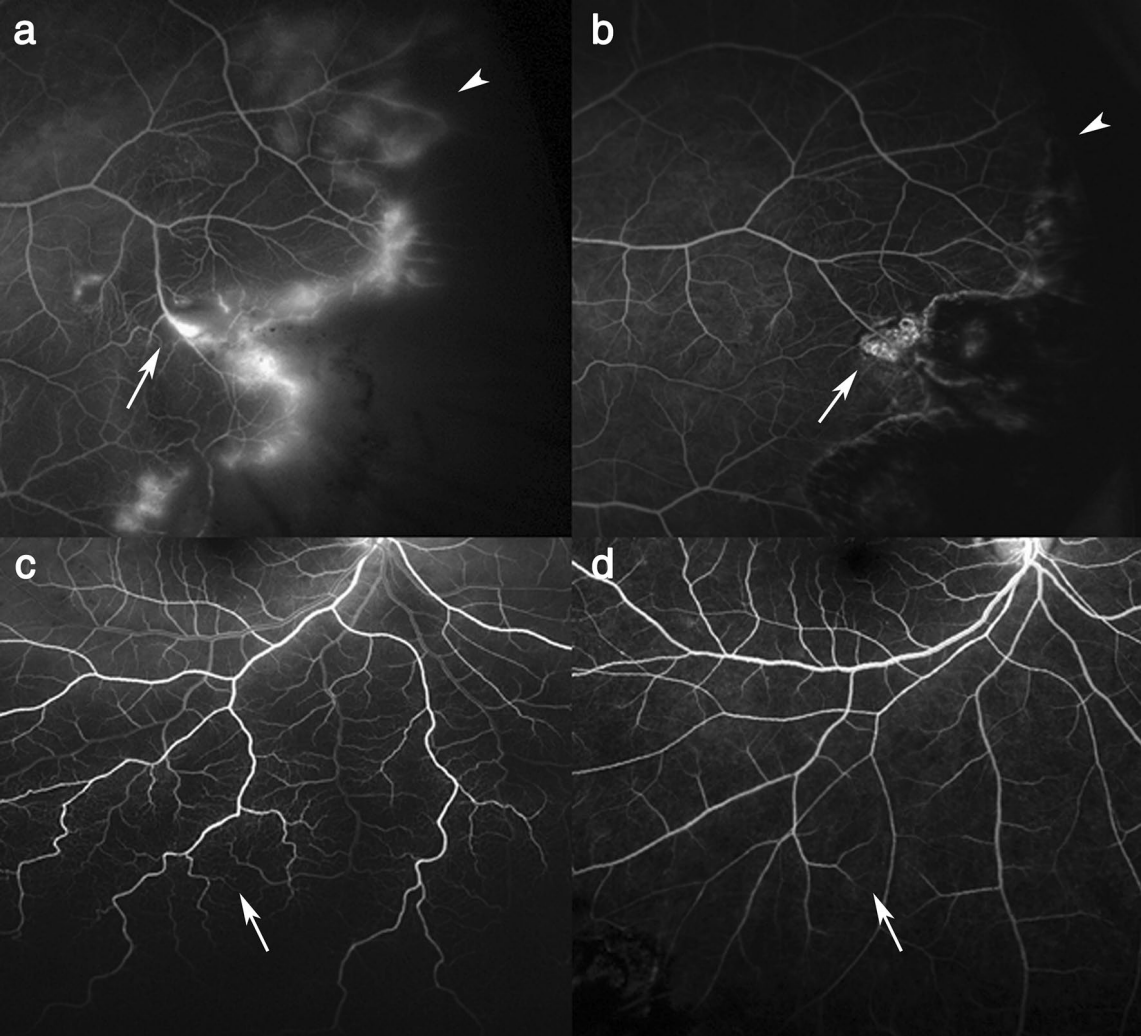
Improvement of vascular abnormalities shown in detached retina after reattachment. All images were taken using ultra-widefield fluorescein angiography. Left column (**a** and **c**) images show vascular abnormalities before surgery, while right column (**b** and **d**) images show improvement after reattachment. Neovascularization (arrow) and paravascular leakage (arrowhead) disappeared after reattachment (**a** and **b**). Previously tortuous vessels (arrow) and venous dilation were reversed after reattachment (**c** and **d**).

Unlike OCTA, UWF FA could evaluate not only macular vasculature but also peripheral vascular abnormalities in RRD. UWF FA was performed preoperatively in all 52 eyes and postoperatively in 46 eyes. Several peripheral vascular abnormalities, including vascular tortuosity, diffuse leakage and nonperfusion, were revealed in the detached retinal area (Table 3, Fig. 4). Diffuse vascular leakage with tortuosity and dilation in the detached retina was observed in all (100%) eyes. Nonperfusion, capillary stasis and arteriovenous transit time delay were commonly noted, although the eyes did not show any vascular obstructive diseases. Interestingly, after reattachment, the vascular abnormalities, even neovascularization, showed partial or even total improvement and regression (Fig. 5).

**Table 3:** Perfusion impairment and vascular abnormalities detected in preoperative ultra-widefield fluorescein angiography in rhegmatogenous retinal detachment.

	RRD (n = 52)
Vascular tortuosity	52 (100%)
Venous dilation	52 (100%)
Diffuse paravascular leaking	52 (100%)
Peripheral nonperfusion	46 (88%)
Marginal leaking	43 (83%)
Capillary stasis	42 (80%)
Delayed arteriovenous transit	36 (69%)
Focal nonperfusion	22 (42%)
Neovascularization	4 (7%)

*RRD* rhegmatogenous retinal detachment.

## Discussion

The retinal vascular system is an end-artery system that does not anastomose with other blood vessels^19^. Using OCTA and UWF-FA, this study provides clinical evidence that the circulation of the retina is damaged at the macula as well as the periphery in RRD. In addition, we elucidated the different impacts on macular circulation in macula-off RRD and age-matched CSC eyes, which could explain a possible mechanism of rapid photoreceptor damage in macula-off RRD. To the best of our knowledge, this is the first longitudinal evaluation of RRD before and after reattachment using UWF FA.

Macular perfusion impairment in RRD and its association with final visual acuity were reported in several previous studies using OCTA^11,12^. In the present study, we observed a negative correlation between the duration of macular detachment and macular capillary flow, which was also associated with photoreceptor disruption and vision loss.

In general, choriocapillaris and choroidal flow have been considered a main oxygen and nutrient supplier for photoreceptors. Nevertheless, recent studies on retinal vasculopathies, including diabetic retinopathy or retinal vein occlusion, have revealed that the DCP vasculature also plays an important role in photoreceptor metabolic support^19–23^. In the detached retina, the role of DCP circulation in photoreceptor viability becomes vital because of the lack of supply from choriocapillaris and choroidal circulation. Therefore, DCP destruction of RRD is critical for macular photoreceptor damage.

It is intriguing that the damage to retinal circulation in macula-off RRD had a positive correlation with the duration of detachment. According to a previous report, vascular abnormalities at the detached retina could be improved after reattachment^24^. This finding emphasizes that prompt reattachment is crucial for preventing further macular vasculature devastation in macula-off RRD. Case reports describing peripheral nonperfusion and neovascularization as long-term complications of RRD also support the importance of early reattachment^4–8^.

We hypothesized the pathological mechanism of macular retinal vascular impairment in relation to its hemodynamics. Retinal circulation is in laminar flow at the attached retina between the vitreous and RPE. Once detached, increased tissue resistance due to the fluctuation of the detached soft retina can cause turbulence and disturb capillary blood flow (See Supplementary Fig. S2 online). Because blood flow and shear stress promote the survival of endothelial cells^25,26^, low blood flow in the detached retina can lead to further closure of the capillary lumen, formation of ghost vessels and eventual regional nonperfusion. Myopia, which is a common predisposition in RRD, can accelerate circulatory compromise due to preexisting peripheral hypoperfusion^27^.

In contrast, retinal detachment in CSC is relatively subtle, and peripheral retinal circulation remains normal, so it may not be sufficient for inducing changes in intraretinal fluid dynamics. Reattachment of the retina could not only recover the choroidal supply but also improve retinal perfusion by tightening tissue to sustain the capillary lumen and reducing tissue resistance. A case in our study shows the rapid reversibility of vascular insufficiency in scleral buckle cases even though the retina is not completely reattached (See Supplementary Fig. S3 online). This observation indicated that the survival of retinal tissue and photoreceptors could be achieved by restoring retinal capillary circulation without recovering choriocapillaris supply.

There are various explanations that can account for the difference between RRD and CSC other than retinal capillary perfusion. The difference in height of SRFs can have a bearing on the oxygen transmission. A large amount of SRF in RRD can make it difficult to transmit oxygen from the choriocapillaris. In CSC, however, SRF height and OCTA parameters did not show a significant relationship (Supplementary Fig. S1). The different origins of the SRF can also influence photoreceptor oxygenation. Exudates from the choriocapillaris and choroid that carry high oxygen pressure in CSC can maintain photoreceptor function, while SRF originating from liquefied vitreous with low oxygen in RRD cannot maintain photoreceptor health. Furthermore, an abundance of toxic enzymes such as matrix metalloproteinase (MMP) in subretinal fluid, which had been considered to be derived from vitreous fluid, was related to photoreceptor damage in RRD^28,29^. In the experimental mice model, zinc dyshomeostasis induced by hypoxic insult resulted in MMP activation and accumulation in SRF of induced RRD, indicating that hypoxia plays a critical role in toxic enzyme accumulation and photoreceptor damage in RRD^30^.

This study has several limitations. In addition to the retrospective design and relatively small sample size, the follow-up period and the tamponade material were diverse in each case. A longer follow-up period and uniform tamponade can provide more concise information. Additionally, preoperative OCTA could not be acquired and analyzed in RRD because of extensive segmentation artifacts in the detached retina. Treatment modalities in patients with CSC varied from spontaneous resolution to photodynamic therapy. The number of patients with each treatment modality was insufficient to compare OCTA results or visual outcomes. We also considered that the influence of the treatment modality on OCTA results or visual outcomes was minimal. Finally, we could not deduce the precise cause of the perfusion differences between RRD and CSC in the present study. SRF acquisition and analysis of its characteristics in both diseases can be helpful, but it could not be done in this retrospective study. Future prospective studies that control possible confounding systemic factors, including diabetes or hypertension, can provide more information on the subject.

In conclusion, while both macula-off RRD and CSC cause macular capillary flow disturbance, RRD induces more rapid and severe flow damage, especially in DCP. Macular capillary loss of flow, which was associated with photoreceptor disruption, correlated with duration of detachment. Peripheral hypoperfusion, venous stasis, and associated vasculopathies present at the detached retina can be improved after reattachment. Thus, early reattachment and reperfusion are required for minimizing macular capillary damage and for the prevention of visual impairment in macula-off RRD.

## Supplementary Information

Supplementary Figure S1.

Supplementary Figure S2.

Supplementary Figure S3.
