# Supplementary material for: Rhegmatogenous retinal detachment induces more severe macular capillary changes than central serous chorioretinopathy
Source: Sci Rep. 2022 Apr 29;12:7018. doi: 10.1038/s41598-022-11062-6 (PMC9054837; doi:10.1038/s41598-022-11062-6)
Supplement: Supplementary file 1 — Supplementary Figure S1. [file 41598_2022_11062_MOESM1_ESM.pdf]

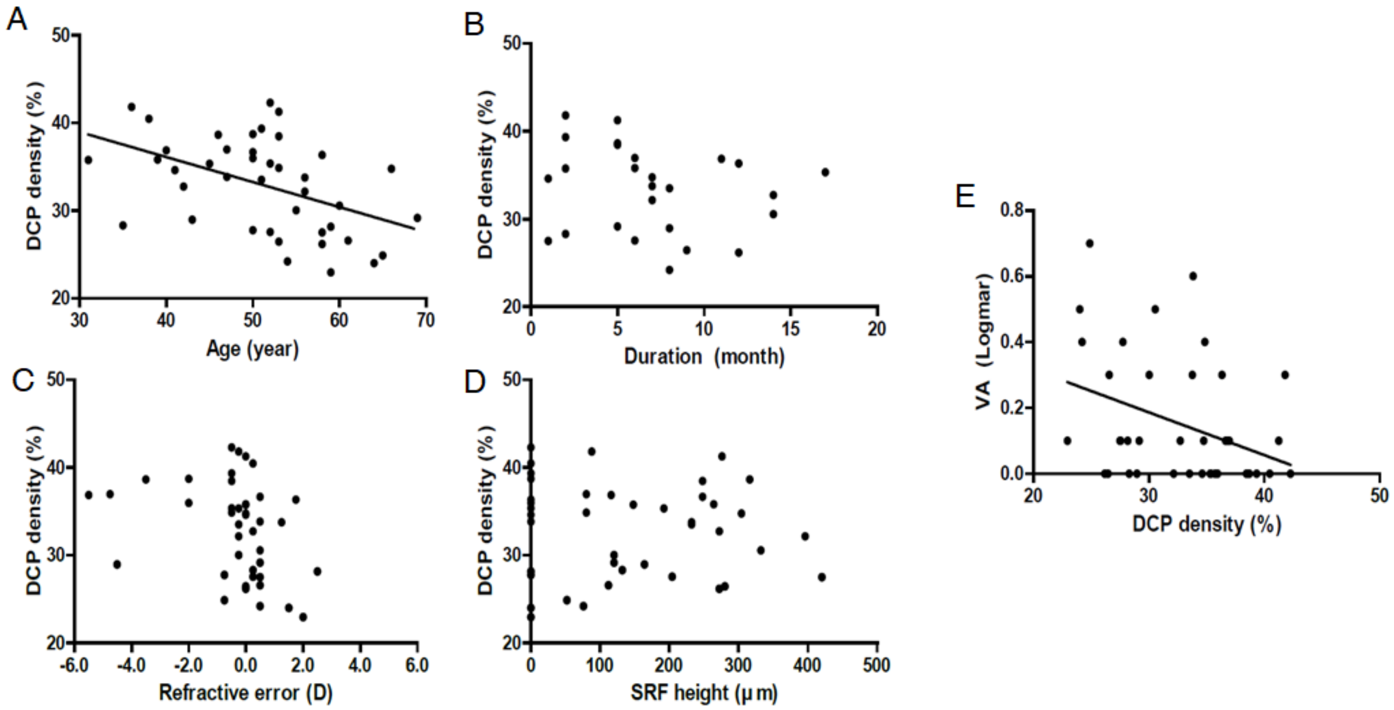

Supplementary Figure S1. Relationships between different parameters in central serous chorioretinopathy (CSC).

(A) Deep capillary plexus (DCP) density was negatively correlated with age. Because we found this relationship, we selected age-matched groups of eyes with rhegmatogenous reintal detachment and CSC

(B) Unlike RRD, CSC did not show significant relationship between DCP density and duration of macular detachment.

(C and D) DCP density did not show any relationship with refractive error or subretinal fluid height.

(E) Visual acuity (logMAR) showed a negative correlation to DCP density.
