# Supplementary material for: Rhegmatogenous retinal detachment induces more severe macular capillary changes than central serous chorioretinopathy
Source: Sci Rep. 2022 Apr 29;12:7018. doi: 10.1038/s41598-022-11062-6 (PMC9054837; doi:10.1038/s41598-022-11062-6)
Supplement: Supplementary file 2 — Supplementary Figure S2. [file 41598_2022_11062_MOESM2_ESM.pdf]

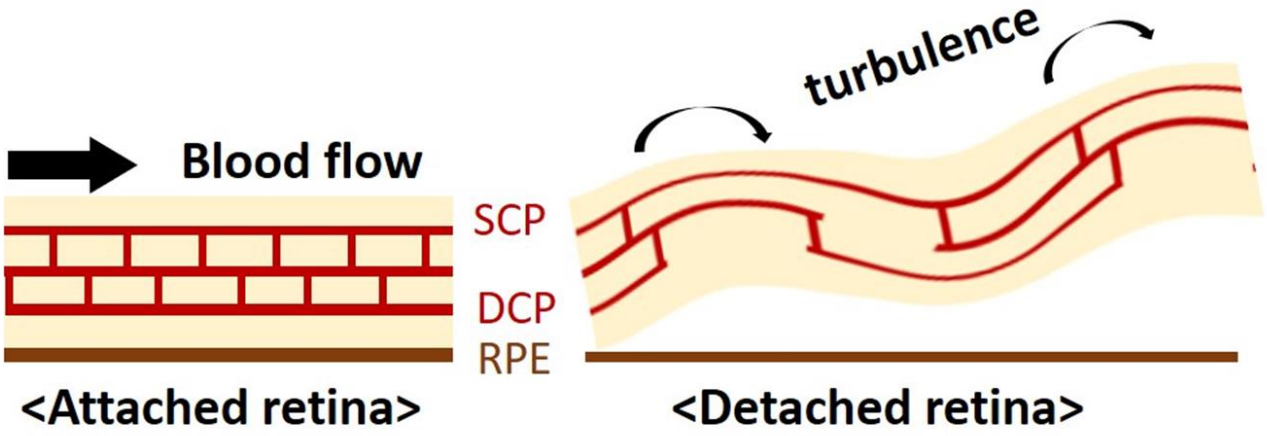

Supplementary Figure S2. Schematic drawing of dynamics of blood flow in attached and detached retina.

Retinal circulation is in calm and laminar flow at the attached retina between the vitreous and RPE. Once detached, increased tissue resistance due to the fluctuation of the detached soft retina can cause turbulence in laminar flow and disturb capillary blood flow.
