# Supplementary material for: Rhegmatogenous retinal detachment induces more severe macular capillary changes than central serous chorioretinopathy
Source: Sci Rep. 2022 Apr 29;12:7018. doi: 10.1038/s41598-022-11062-6 (PMC9054837; doi:10.1038/s41598-022-11062-6)
Supplement: Supplementary file 3 — Supplementary Figure S3. [file 41598_2022_11062_MOESM3_ESM.pdf]

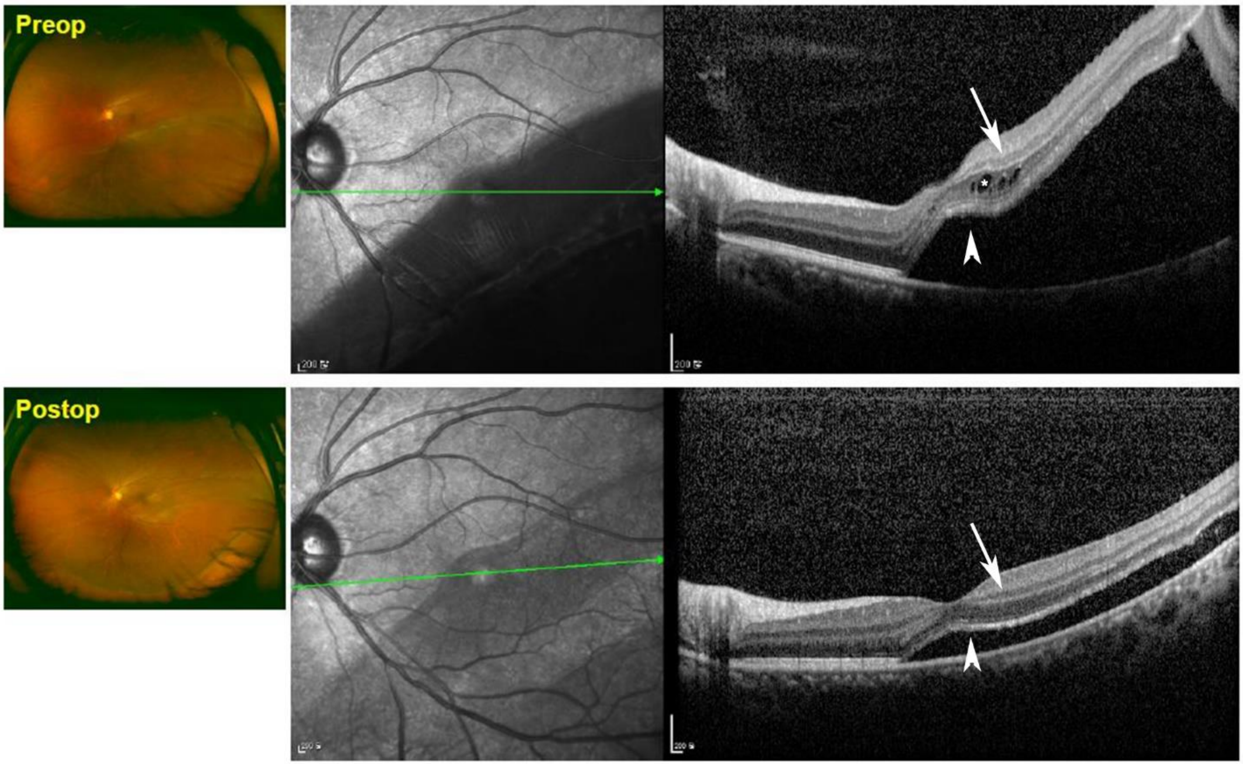

Supplementary Figure S3. Representative case of incomplete retinal reattachment. Upper row (A and B) are images of preoperative status, while lower row (C and D) are images after performing scleral buckling operation. Initially, patient's vision was 20/32, and OCT revealed that macula-off RRD with prominent middle limiting membrane sign (arrow), elongated photoreceptor outer segment, ellipsoid zone disruption(arrowhead) and intraretinal cysts (asterisk). After the operation, retina was flattened down but small amount of subretinal fluid remained. However, preoperatively shown signs of retinal vascular insufficiency on OCT disappeared and improved although the complete retinal reattachment was not achieved (arrow and arrowhead). Improvement of macular edema, ellipsoid zone integrity and visual acuity (20/20) could be considered due to alteration of dynamics of retinal blood flow with improvement of retinal perfusion.
